# 3D assessment of ear morphology

**DOI:** 10.1016/j.jobcr.2023.08.001

**Published:** 2023-08-04

**Authors:** Meleti Venkata Sowmya, Divya Mehrotra, Shadab Mohammad, R.K. Singh, Arunesh Kumar Tiwari, Ravi Katrolia, Shivani Sharma Yogesh

**Affiliations:** Department of Oral and Maxillofacial Surgery, King George Medical University, Lucknow, India

**Keywords:** Ear morphology, Stereophotogrammetry, Anthropometry

## Abstract

**Introduction:**

Auricular reconstruction is a technically challenging and aesthetically demanding procedure as the ear has a complex anatomy. Anthropometry aids in achieving aesthetic ear reconstruction. We considered that implication of stereophotogrammetric technology will lead to a better understanding of human ear morphology.

**Material and methods:**

A cross-sectional study was conducted in our institutional OPD in a tertiary health care centre in the Northern part of India.400 people were chosen based on selection criteria. facial scans were done for 3D pictures using Canfield VECTRA® H2 3D imaging device. Study variables were assessed after marking landmarks on the 3D-generated auricular image of an individual.

**Discussion:**

This study consisted of 55.5% males and 44.5% females belonging to the age group of 5–25 years (30.3%), 26–40 years (38.8%) and>40 yr (31.0%). Out of 400 cases, the majority had; oval shaped auricle, normally rolled helix, square earlobe; knob shaped tragus. The attached type of earlobe attachment was more in the right auricle (37%) and the partial attachment ear lobe was more in the left side auricle (35.5%). Darwin's tubercle showed more proportion in the case of males. The mean length and width of the auricle & attachment length are higher in males compared to females. Ear Angulation is highest among females.

**Conclusion:**

Assessment of ear morphology using technologically sound methods like stereophotogrammetry paves the way for a more quick, reliable and easy-to-use method for understanding ear morphology. Precise assessment of ear morphology using stereophotogrammetry helps in providing more cosmetic and acceptable ear restoration.

## Graphical abstract

**Figure:**
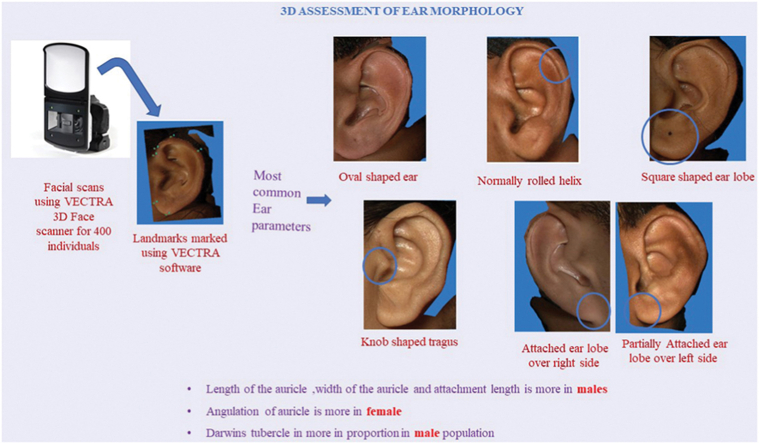

## 1 Introduction

Since antiquity, humans have graded the attractiveness of a person based on facial proportions and features. A well-proportioned face is considered aesthetic. There are numerous studies which investigated facial morphology concerning ethnic variations, age and gender differences, symmetry, and attractiveness.The ear is a dominant landmark of the human face and deformities of the ear can be mutilating and may have profound psychological effects.

The study of ear morphology helps in understanding and correcting ear deformities and finds its way into the field of forensics for the identification of criminals and deceased, determining the sex and age of an individual.

Auricle defects may be congenital malformations like microtia, anotia, atresia (the external auditory canal is closed) or acquired auricular defects. Auricular reconstruction is a technically challenging and aesthetically demanding procedure as the ear has a complex anatomy. Anthropometry aids in achieving aesthetic ear reconstruction, but the techniques of anthropometry range from simple ear measurements made using a vernier calliper to three-dimensional surface imaging based on stereophotogrammetric analysis. Conventional modes of ear morphology measurements and analysis may lack accuracy. Another shortcoming in the existing literature is that most of the existing studies concentrate on measurements over the entire face while only a few focus on the specific parts of the face, e.g., the ear.^1^

The most common ear restoration technique involves the creation and implantation of an autologous construct fashioned by hand from costal cartilage, yielding acceptable aesthetic outcomes with a durable and compatible implant. This process is demanding given the intricacy of repair, significant psychosocial sequelae, and potential complications including pneumothorax, infection, exposure of the implant, and scarring. Although porous polyethylene implants can provide good aesthetic outcomes, studies report higher complications like extrusion and infection. 3D printing may lessen the technical complexity of autologous implants and produce high-fidelity, biocompatible implants but with challenges like framework contraction/distortion, poor long-term outcomes, and high regulatory burden.3D anthropometric assessment of ear morphology will provide a database which helps in ear reconstruction by digital means.

Hence, this study was conducted to assess the 3D morphology of the human ear in the North Indian population using stereophotogrammetric analysis by Canfield VECTRA® H2 (Canfield Imaging, Parsippany, NJ, USA) 3D imaging device and to create a database of measurements of the human ear in different age groups.

## 2 Objectives of the study

• **To assess Ear morphology in the North Indian population.**
• **To create a database of measurements of the human Ear.**

## 3 Material and methods

We conducted a cross-sectional study in our institutional OPD in a tertiary health care centre in the Northern part of India from January 2022 to June 2022, after obtaining institutional ethical clearance and informed consent from the participant, abiding by the rules of declaration of Helsinki. **The sample size was determined based on the average number of individuals reported to our departmental OPD (study population of 6708) during a 6 months' time .A confidence level of 95% and confidence interval of 5% was choosen.** A sample size of 400 was obtained. **The** sample population was grouped into 3 categories based on age ranges; 5–25 years, 26–40years and >40 years.

### 3.1 Inclusion criteria

1. Normal healthy individuals of both sexes of age greater than 5 years (General population who reported to our departmental OPD and not having any ear defects)
2. Individuals belonging to North Indian ethnicity.

### 3.2 Exclusion criteria

1. Not consenting
2. Infants and children younger than 5 years of age
3. People with ear deformities (either acquired or congenital).

### 3.3 Methodology

Demographic details of each patient were recorded on a set proforma designed for this study. After taking consent from the patient, facial scans were done for 3D pictures for each patient using Canfield VECTRA® H2 (Canfield Imaging, Parsippany, NJ, USA) 3D imaging device. This device is based on stereophotogrammetry technology with a capture time of 2.0 ms. Stereophotogrammetry is a surface imaging technology that involves estimating the 3D coordinates of points on a surface employing measurements made in 2 or more photographic images taken from different positions.

**Participant preparation: The participant was instructed to remove all jewellery and clothing near the neck and to wipe out oils, sweat or any make-up before facial imaging. The patient's hair was secured away from the face, ears and neck with a hair band.** The participant was asked to stand completely still on the positioning mat throughout the 3 face captures while the photographer moved. The participant was instructed to keep the eyes open, gaze fixed straight ahead, not look up or down, mouth closed and relaxed facial expression. The sequence of image capturing was right profile, frontal view followed by left profile. For right and left side captures the operator stood at 45° from the direction the patient was facing. The camera was held at the patient's chest level, about 12 inches (30 cm) below mid-face, and angled upwards. The red dots were aimed at the middle of the patient's cheek (intersection of imaginary lines from the lateral canthus and the upper lip). For the frontal view, the operator stood directly in front of the patient. The camera was raised to a level with the patient's face ensuring that the camera was straight and not angled. The red dots were aimed between the upper lip and nose, at the mid-line of the patient's face. The red dots converged to a single point by adjusting the camera distance from the patient. The image was captured.

The raw images captured in the camera were imported into the VECTRA® software. The images were stitched to get a 3D image of the patient. Such images were captured for each patient to assess the morphology of the ear.

**Fig. 1:**
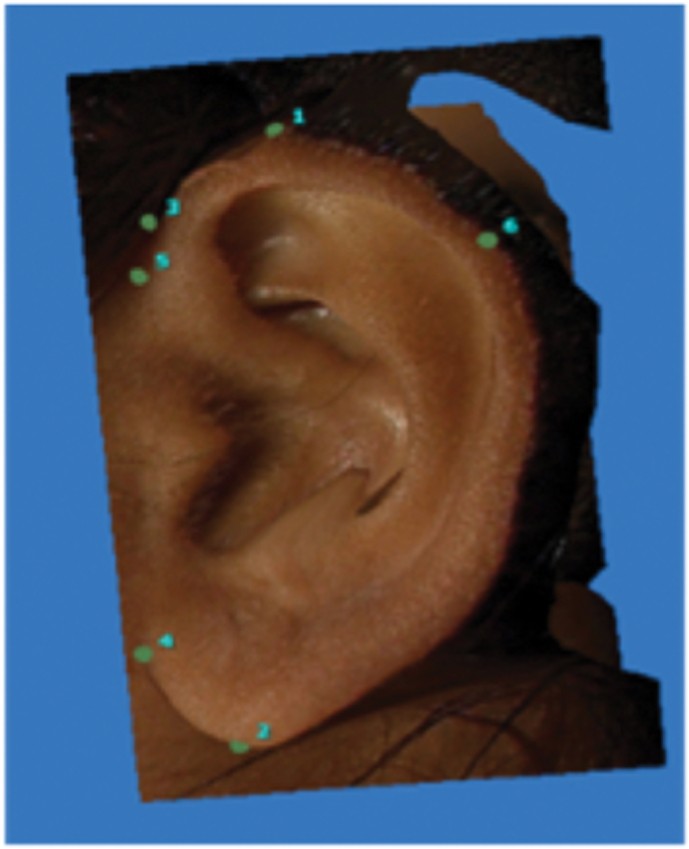
Landmarks marked over the Ear.

The following landmarks were marked on the 3D stitched images of an individual for further analysis (Fig. 1)^2^

1. **Super-aurale (SupAu):** The most cranial point of the auricle with the head positioned according to the Frankfurt horizontal plane.
2. **Sub-aurale (sba):** The most caudal point located on the earlobe with the head positioned according to the Frankfurt horizontal plane. If the "attached earlobe" appearance is present the point is identical to the otobasion inferius point.
3. **Post-aurale (pa):** The most dorsal point of the ear with the head positioned according to the Frankfurt horizontal plane.
4. **Otobasion superius (obs): Cranial** point of attachment of ear to head
5. **Otobasion inferius (obi):** Caudal point of attachment of ear to the cheek
6. **Preaurale (pra):** the Most ventral point of the ear.

**Fig. 2:**
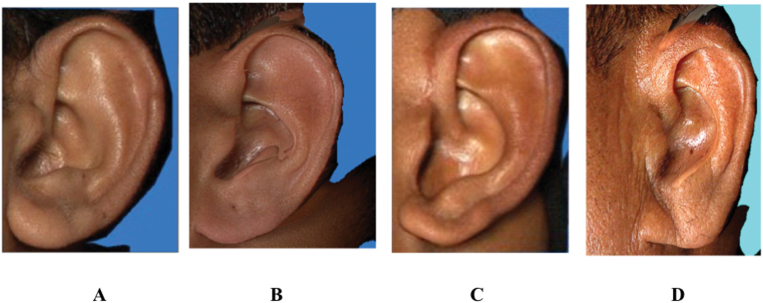
Images showing the shape of ear • 2A: Round shape • 2B: Oval shape • 2C: Triangular shape • 2D: Rectangular shape.

**Fig. 3:**
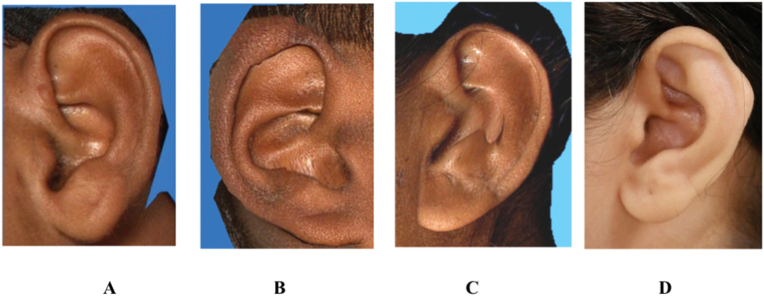
Images showing the Types of Ear helix • 3A: Normally rolled helix • 3B: Wide helix overing scapha • 3C: Flat helix • 3D: Concave marginal helix.

**Fig. 4:**
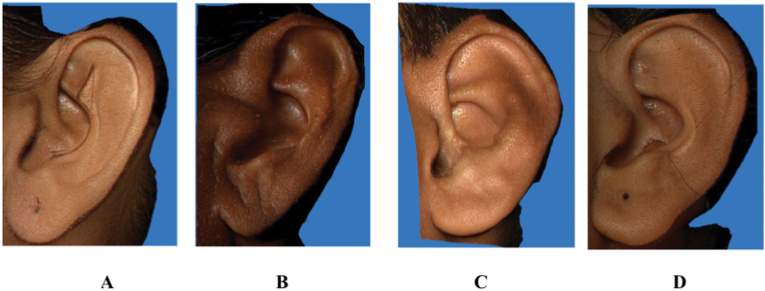
Images showing the Types of Ear lobe • 4A: Tongue Shape • 4B: Triangular Shape • 4C: Arched Shape • 4D: Square Shape.

**Fig. 5:**
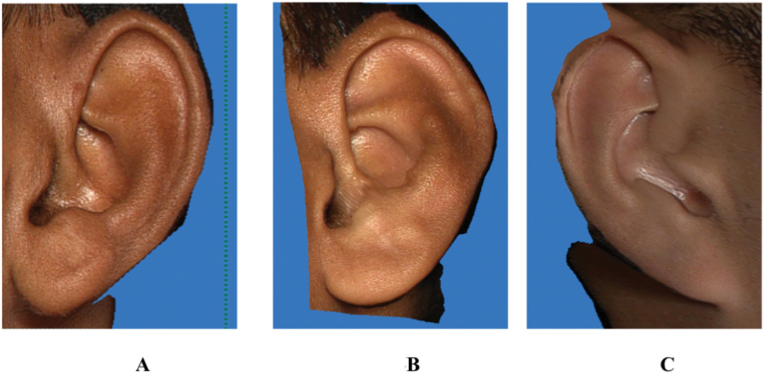
Images showing attachment types of Ear lobe • 5A: Free Earlobe • 5B: Partially attached Earlobe • 5C: Attached Earlobe.

**Fig. 6:**
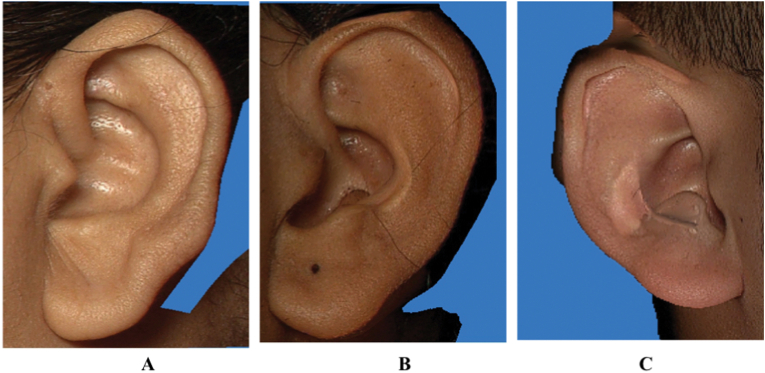
Images showing shapes of Tragus • 6A: Knob shape • 6B: Round shape • 6C: Long.

**Fig. 7:**
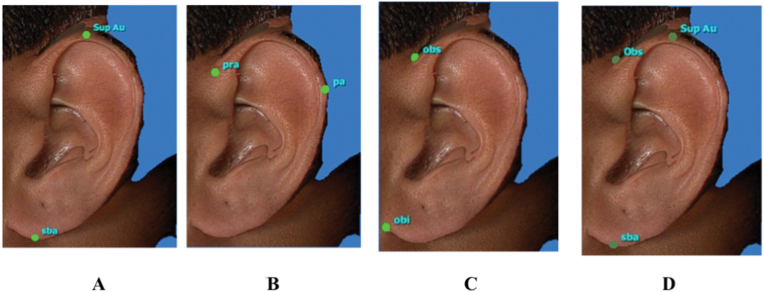
Images showing the study variables marked • 7A: Length of the ear • 7B: Width of the ear • 7C: Attachment length of the ear • 7D: Angulation of the ear.

### 3.4 Study variables

1) Shape of the ear: Round, Oval, Triangular, Rectangular(Fig. 2)
2) Shape of the helix: Normally rolled, Wide covering scapha, Flat, Concave marginal (Fig. 3)
3) Shape of the earlobe: Tongue, Triangular, Arched, Square (Fig. 4)
4) Attachment of the earlobe: Free, Partially attached, Attached(Fig. 5)
5) Shape of the tragus: Knob, Round, Long (Fig. 6)
6) Darwin's tubercle: Darwin's tubercle refers to a congenital prominence usually found on the posterior part of the helix of the ear
7) Ear inclination (Sup Au- Sba- Obs): Ear angulation is considered as the angle formed between Super-Aurale (Sup-Au), Sub-Aurale (Sba) and Otobasion Superious (Obs).
8) Length of the auricle (Sup Au- sba)- distance from Super Aurale to Sub aurale
9) Width of the auricle (Pa-Pra): Distance between post-aurale and pre aurale
10) Attachment length (O Sup-O Inf): Distance between otobasion superius to otobasion inferius(Fig. 7).

**Landmarks were marked on the 3D-generated ear image** and the distance between the landmarks was measured in a straight line with the help of in-built software programmed in the VECTRA® analysis module, an extension of the VECTRA® Face Sculptor. The study variables were recorded for each ear image and Values were tabulated. All statistical analyses were performed using Statistical Package for Social Sciences (SPSS) 16.0 Windows software. Derived data was used to assess the significance of the association between study variables with the age and gender of an individual. Unpaired *t*-test, ANOVA, chi-square test and multivariate regression analysis were used wherever appropriate for statistical analysis of the data to derive results.

## 4 Discussion and results

Aesthetic rehabilitation of the ear requires sound knowledge regarding ear morphology and how it varies with the age and gender of an individual. Various studies have been conducted to date for understanding human ear morphology, employing different tools like vernier callipers, model casts, laser scanning and digital imaging technology. Stereophotogrammetrical analysis helps in the precise assessment of soft tissues including finer details. 3D photogrammetry has the potential value of statistical shape analysis in assessing ear morphology.

This study consisted of 222 (55.5%) males and the rest 178 (44.5%) females belonging to the age group of 5–25 years (30.3%), 26–40 years (38.8%), >40 years (31.0%). According to our study Oval shaped ear was the commonest among both sexes on both sides which is as per the studies of **Singh P et al(2009**^**)**^^3^^**,**^ **Verma P et al (2016)**^4^ **Krishan K et al (2019**^**)**^^5^^**,**^ **Purkait et al (2007)**^6^ But According to **Rani D et al (2020)**^7^ round shaped ears were most common among females. Both right and left ears showed significant association with age with rectangular and round shaped ears found more in proportion in older ages. No such association of ear shape was found with the sex of an individual in the current study (Table 1).

**Table 1:** Distribution of ear shape by age and gender.

Side	Variable		Ear Shape								chi sq	p-value
			Oval		Recta-ngular		Round		Trian-gular			
			No	%	No	%	No	%	No	%		
Right Ear	Age	5–25 yr	90	74.4	14	11.6	8	6.6	9	7.4	17.24	**0.008**
		26–40 yr	97	62.6	18	11.6	15	9.7	25	16.1		
		>40 yr	63	50.8	26	21.0	13	10.5	22	17.7		
	Sex	Male	137	61.7	37	16.7	17	7.7	31	14.0	2.66	0.446
		Female	113	63.5	21	11.8	19	10.7	25	14.0		
Left Ear	Age	5–25 yr	102	84.3	10	8.3	2	1.7	7	5.8	24.60	**<0.001**
		26–40 yr	111	71.6	11	7.1	7	4.5	26	16.8		
		>40 yr	79	63.7	21	16.9	11	8.9	13	10.5		
	Sex	Male	158	71.2	25	11.3	11	5.0	28	12.6	1.04	0.791
		Female	134	75.3	17	9.6	9	5.1	18	10.1		

Normally rolled helix is the most common type with an incidence of 80% over the right side and 86% over the left side. This was similar to some previous studies.^3^^,^^6^^,^^7^Wide helix covering scapha has the lowest incidence among all. There is a significant correlation showing that concave marginal-shaped helix was more common in the case of younger age groups and the flat-shaped helix was more among the right ear of males (Table 2).

**Table 2:** Distribution of shape of the Helix by Age and Gender.

Side	Variable		Helix								chi sq	p-value
			Concave Marginal		Flat		Normally Rolled		Wide Covering Scapha			
			No	%	No	%	No	%	No	%		
Right Ear	Age	5–25 yr	11	9.1	5	4.1	103	85.1	2	1.7	35.97	**<0.001**
		26–40 yr	7	4.5	23	14.8	125	80.6	0	0.0		
		>40 yr	0	0.0	32	25.8	92	74.2	0	0.0		
	SEX	Male	8	3.6	44	19.8	170	76.6	0	0.0	11.84	**0.008**
		Female	10	5.6	16	9.0	150	84.3	2	1.1		
Left Ear	Age	5–25 yr	4	3.3	4	3.3	108	89.3	5	4.1	26.16	**<0.001**
		26–40 yr	4	2.6	15	9.7	135	87.1	1	0.6		
		>40 yr	0	0.0	23	18.5	101	81.5	0	0.0		
	SEX	Male	4	1.8	26	11.7	190	85.6	2	0.9	2.00	0.573
		Female	4	2.2	16	9.0	154	86.5	4	2.2		

The shape of the Ear lobe showed that the square-shaped ear lobe was commonest both in the right (33.5%) and left sides (36.5%) which was consistent with the studies of **Sharma et al (2007)**.^8^ The triangular-shaped ear lobe is of least percentage which is in line with the study of **Rani D et al (2020)**.^7^ There is a significant association showing arched type ear lobes being more in proportion in the case of younger age groups on the right side and Tongue-shaped ear lobes on the left side. Triangular ear lobes show significant association with the male sex over the right side (Table 3). Cartilage free nature of the ear lobe leading to sagging or expansion of extracellular matrixes explains the change in length and shape of the ear lobe with age.^9^^,^^10^

**Table 3:** Distribution of earlobe by age and gender.

Side	Variable		Earlobe								chi sq	p-value
			Arched		Square		Tongue		Trian-gular			
			No	%	No	%	No	%	No	%		
Right Ear	Age	5–25 yr	48	39.7	38	31.4	31	25.6	4	3.3	25.91	**<0.001**
		26–40 yr	51	32.9	46	29.7	46	29.7	12	7.7		
		>40 yr	21	16.9	50	40.3	33	26.6	20	16.1		
	Sex	Male	67	30.2	78	35.1	49	22.1	28	12.6	12.98	**0.005**
		Female	53	29.8	56	31.5	61	34.3	8	4.5		
Left Ear	Age	5–25 yr	36	29.8	40	33.1	37	30.6	8	6.6	16.44	**0.012**
		26–40 yr	52	33.5	55	35.5	39	25.2	9	5.8		
		>40 yr	18	14.5	51	41.1	40	32.3	15	12.1		
	Sex	Male	62	27.9	74	33.3	70	31.5	16	7.2	3.25	0.355
		Female	44	24.7	72	40.4	46	25.8	16	9.0		

The attached type of ear lobe attachment is more common in the case of the right auricle and the partially attached type is more common in the left auricle. Earlier studies on ear lobe attachment showed that attached type ear lobe attachment is more in percentage compared to other types.^4^^,^^5^^,^^11^ But according to few studies free ear lobes are more in frequency.^3^^,^^6^^,^^12^, ^13^, ^14^ According to **Rani D et al (2020)**^7^ partially attached ear lobule attachment is more common followed by attached ear lobule and free ear lobule(Table 4).

**Table 4:** Distribution of Ear lobe attachment by Age and Gender.

Side	Variable		Attachment						chi sq	p-value
			Attached		Partially Attached		Free			
			No	%	No	%	No	%		
Right Ear	Age	5–25 yr	59	48.8	37	30.6	25	20.7	28.12	**<0.001**
		26–40 yr	65	41.9	41	26.5	49	31.6		
		>40 yr	24	19.4	54	43.5	46	37.1		
	Sex	Male	82	36.9	77	34.7	63	28.4	0.87	0.648
		Female	66	37.1	55	30.9	57	32.0		
Left Ear	Age	5–25 yr	41	33.9	45	37.2	35	28.9	18.21	**0.001**
		26–40 yr	63	40.6	47	30.3	45	29.0		
		>40 yr	22	17.7	50	40.3	52	41.9		
	Sex	Male	74	33.3	78	35.1	70	31.5	0.88	0.645
		Female	52	29.2	64	36.0	62	34.8		

Tragus of the ear showed that the knob-shaped tragus has the highest proportion of occurrence with 46% over the right and 46.5% over the left side. The shape of the Right tragus showed no significant association with the sex of an individual, but the left tragus showed a significant association between the knob-shaped tragus and middle age group (26–40 years) (Table-5).

**Table 5:** Distribution of tragus by age and gender.

Side	Variable		Tragus						chi sq	p-value
			Knob		Long		Round			
			No	%	No	%	No	%		
Right Ear	Age	5–25 yr	50	41.3	40	33.1	31	25.6	8.26	0.083
		26–40 yr	85	54.8	41	26.5	29	18.7		
		>40 yr	49	39.5	41	33.1	34	27.4		
	Sex	Male	104	46.8	59	26.6	59	26.6	4.60	0.100
		Female	80	44.9	63	35.4	35	19.7		
Left Ear	Age	5–25 yr	52	43.0	38	31.4	31	25.6	9.59	**0.048**
		26–40 yr	84	54.2	32	20.6	39	25.2		
		>40 yr	50	40.3	30	24.2	44	35.5		
	Sex	Male	96	43.2	53	23.9	73	32.9	4.75	0.093
		Female	90	50.6	47	26.4	41	23.0		

Darwin's tubercle was present in 29% of the sample population in the right ear and 28% in the case of the left ear. Earlier studies by **Krishan K et al (2019)**^5^ reported an incidence of 46–67.8%. Left ear Darwin's tubercle showed significant association with the age of an individual by being more in the younger age group. It also showed more proportion in the case of males. Rubio **O et al (2015)**^15^ concluded that Darwin's tubercle shows neither sexual dimorphism nor relation with the age of an individual (Table 6).

**Table 6:** Distribution of Darwin's tubercle by age and gender.

Side	Variable		Darwin's tubercle				chi sq	p-value
			Absent		Present			
			No	%	No	%		
Right Ear	Age	5–25 yr	79	65.3	42	34.7	3.00	0.223
		26–40 yr	112	72.3	43	27.7		
		>40 yr	93	75.0	31	25.0		
	Sex	Male	149	67.1	73	32.9	3.65	0.056
		Female	135	75.8	43	24.2		
Left Ear	Age	5–25 yr	76	62.8	45	37.2	14.15	**0.001**
		26–40 yr	108	69.7	47	30.3		
		>40 yr	104	83.9	20	16.1		
	Sex	Male	147	66.2	75	33.8	8.28	**0.004**

The length of the auricle was measured from the cranial most point of the pinna to the caudal end of the pinna. It was seen that the length of both ears increases with the increase in age of an individual. The mean right ear length of males and females according to our analysis is 62.22 ± 5.74 mm and 57.18 ± 5.28 mm respectively. The mean left ear length of males and females is 62.31 ± 5.66 mm and 57.07 ± 4.68 respectively. (Table 7).

**Table 7:** Comparison of length with age and gender.

Side	Variable		Length		significance	
			Mean	SD	F/t - value	p-value
Right Ear	Age	5–25 yr	57.65	5.55	30.15	<0.001
		26–40 yr	59.28	4.92		
		>40 yr	63.10	6.60		
	Sex	Male	62.22	5.74	9.04	**<0.001**
		Female	57.18	5.28		
Left Ear	Age	5–25 yr	57.67	5.25	24.90	**<0.001**
		26–40 yr	59.66	4.78		
		>40 yr	62.62	6.58		
	Sex	Male	62.31	5.66	9.94	**<0.001**

The width of the auricle was measured from the Pre-aurale to the Post-aurale. It was seen that the width of the ear also increases with the increase in the age of an individual. The mean width of the right ear in males and females is 30.31 ± 3.57 mm and 29.25 ± 3.12 mm respectively. The mean width of the left ear in males and females is 29.88 ± 3.47 mm and 29.13 ± 2.72 mm respectively. These values are as per the earlier studies on the width of the auricle.^9^^,^^11^^,^^16^^,^^17^ (Table 8)

**Table 8:** Comparison of width with age and gender.

Side	Variable		Width		significance	
			Mean	SD	F/t - value	p-value
Right Ear	Age	5–25 yr	28.49	3.15	18.83	<0.001
		26–40 yr	29.93	2.72		
		>40 yr	31.04	3.95		
	Sex	Male	30.31	3.57	3.11	**0.002**
		Female	29.25	3.12		
Left Ear	Age	5–25 yr	28.41	3.04	14.32	**<0.001**
		26–40 yr	29.68	2.89		
		>40 yr	30.49	3.32		
	Sex	Male	29.88	3.47	2.35	**0.019**
		Female	29.13	2.72		

The values for the mean length and width of the auricle are higher in males compared to females which is supported by the literature.^2^^,^^4^^,^^10^^,^^18^ But according to **Ahmed A et al (2015)**^19^ the mean width of auricle was higher in the case of females than males. According to **Ahmed A et al (2015)**,^19^ ear measurements cannot be used as an effective tool for sex estimation as these values are not only population-specific, but they are also race specific.

The attachment length of the ear is the total length of the ear which is attached to the face. This is the least recognized parameter of the ear in literature, but according to our study, this parameter will give additional data regarding ear morphology and may find a way in forensic sciences for human identification. The mean attachment length of the right ear according to the current study among males and females are 50.25 ± 5.28 mm and 44.09 ± 5.25 mm respectively. The mean attachment length of the left ear according to the current study among males and females are 50.18 ± 5.18 mm and 43.52 ± 4.87 mm respectively. The attachment length of both ears was increased with an increase in age and is higher among males (Table–9).

**Table 9:** Comparison of attachment length with age and gender.

Side	Variable		Attachment Length		Significance	
			Mean	SD	F/t - value	p-value
Right Ear	Age	5–25 yr	44.97	5.23	38.29	<0.001
		26–40 yr	46.70	5.70		
		>40 yr	51.00	5.78		
	Sex	Male	50.25	5.28	11.63	**<0.001**
		Female	44.09	5.25		
Left Ear	Age	5–25 yr	44.64	5.75	30.52	**<0.001**
		26–40 yr	46.84	5.51		
		>40 yr	50.21	5.66		
	Sex	Male	50.18	5.18	13.12	**<0.001**
		Female	43.52	4.87		

Studies till now on ear angulation were based on the angle formed by the long axis of the ear, either with the Frankfurt horizontal plane or with the facial midline. This angulation helps in identifying the relative position of the ear to the face. But through our study, we calculated the angulation of the ear by measuring the angle between super –aurale, sub –aurale and oto-basion superius. This value helps in identifying the ear position in a more detailed way in relation to the other landmarks of the ear itself making it a reliable parameter while considering 3D ear reconstruction or scaffold preparation. The mean right ear angulation for males and females is 15.26 ± 2.60 and 16.00 ± 2.79 respectively. The mean left ear angulation for males and females is 14.76 ± 2.61 and 16.00 ± 2.98 respectively. The angulation was found to be higher in females (Table–10). **Mohamed et al (2015)**^20^ calculated the angulation between the long axis of the ear and the bridge of the nose and the values are 23.64 ± 3.20 over the right side and 23.04 ± 3.84 over the left side.

**Table 10:** Comparison of angulation with age and gender.

Side	Variable		Angulation		Significance	
			Mean	SD	F/t - value	p-value
Right Ear	Age	5–25 yr	15.54	2.88	0.87	0.419
		26–40 yr	15.43	2.38		
		>40 yr	15.85	2.93		
	Sex	Male	15.26	2.60	−2.75	**0.006**
		Female	16.00	2.79		
Left Ear	Age	5–25 yr	15.52	3.02	0.79	0.453
		26–40 yr	15.10	2.54		
		>40 yr	15.38	3.04		
	Sex	Male	14.76	2.61	−4.41	**<0.001**
		Female	16.00	2.98		

Based on the above interpretations, one can observe that the data derived for the right-side ears is not exacting the left-side ears. Hence, we can conclude that mild asymmetry will be seen between both ears of a single individual. The overall size of the Ear of the male population is comparatively higher compared to females of the same age group. Data derived through this study can help in designing ear scaffolds in case of ear reconstruction either by conventional methods or digital means. It can also find its way into forensic sciences for age and gender identification of an individual based on ear morphology.

Stereophotogrammetry provided valuable quantitative data about the ear landmarks and allowed ease of localizing and reproducibility with no risk of any radiation exposure. But 3D photogrammetry fails to capture fine structures like hair and the presence of hair can lead to distortion of the image. The incorporation of digital technology by using a stereo-photogrammetric surface imaging system combined with computerized software leads to high-level precision and reproducibility in imaging and assessing of human ear.

## 5 Limitations of this study

1) It is a single-centre study so the results cannot be generalized to the entire population.
2) Automatic positioning of landmarks is not available in the current software which may lead to the possibility of interobserver variability while positioning landmarks.

## 6 Conclusion

Assessment of ear morphology using technologically sound methods like stereophotogrammetry paves the way for a more quick, reliable and easy-to-use method for understanding ear morphology. Through this study of 3D assessment of Ear morphology using a Canfield VECTRA H2 imaging device, the following conclusions can be drawn.

1. The oval-shaped ear is a more common ear shape.
2. Normally rolled helix, square ear lobe, Knob shaped tragus is more common in comparison to their counterparts.
3. Darwin's tubercle is more common in Males compared to Females.
4. The attachment length of the ear also should be considered while assessing the morphology of the ear.
5. Angulation of the ear is measured using the angle formed between Sup –aurale, Sub –aurale and Oto-basio superius. According to this study, it is higher in females than males.
6. Precise assessment of ear morphology using stereophotogrammetry helps in providing more cosmetic and acceptable ear restoration.

## Funding

This research did not receive any specific grant from funding agencies in public, commercial or not-for-profit sectors.

## Ethical statement/confirmation of patient's permission

The study was approved by the institutional ethical committee (Ref no-VII-PGTSC-IIA/P21). The procedure was carried out in accordance with **The Code of Ethics of the World Medical Association** (Declaration of Helsinki).

## Consent to participate

Proper consent was taken from the patient and was explained the entire procedure.

## Declaration of competing interest

None.
